# New Concepts in the Development and Malformation of the Arterial Valves

**DOI:** 10.3390/jcdd7040038

**Published:** 2020-09-24

**Authors:** Deborah J. Henderson, Lorraine Eley, Bill Chaudhry

**Affiliations:** Biosciences Institute, Newcastle University, Newcastle upon Tyne NE1 3BZ, UK; lorraine.eley@ncl.ac.uk (L.E.); bill.chaudhry@ncl.ac.uk (B.C.)

**Keywords:** arterial valve, semilunar, outflow cushion, leaflet, EndMT, second heart field, neural crest cells, signalling, bicuspid aortic valve

## Abstract

Although in many ways the arterial and atrioventricular valves are similar, both being derived for the most part from endocardial cushions, we now know that the arterial valves and their surrounding structures are uniquely dependent on progenitors from both the second heart field (SHF) and neural crest cells (NCC). Here, we will review aspects of arterial valve development, highlighting how our appreciation of NCC and the discovery of the SHF have altered our developmental models. We will highlight areas of research that have been particularly instructive for understanding how the leaflets form and remodel, as well as those with limited or conflicting results. With this background, we will explore how this developmental knowledge can help us to understand human valve malformations, particularly those of the bicuspid aortic valve (BAV). Controversies and the current state of valve genomics will be indicated.

## 1. Anatomy, Histology and Nomenclature of the Mature Arterial (Semilunar) Valves

**Figure 1:**
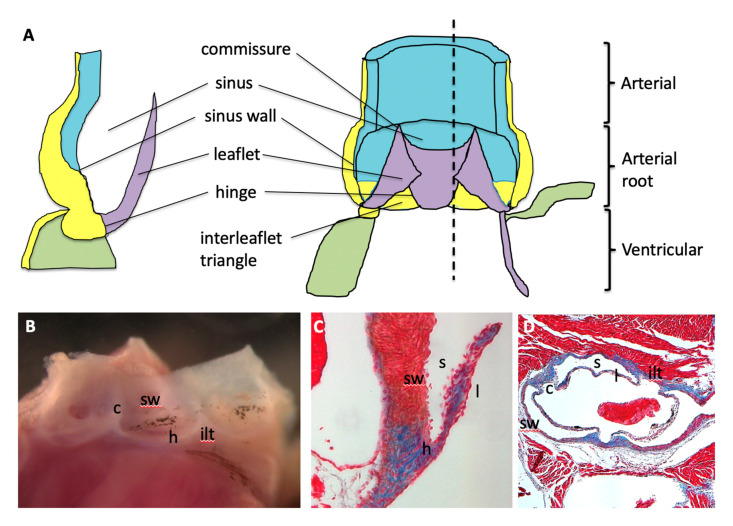
Anatomy of the arterial roots. (**A**) The arterial valve complex is made up of: three moving leaflets; the hinges, the attachment points of the leaflets to the wall; the commissures, the points of apposition of the leaflets close to the wall; the sinuses, pockets that form between the leaflets and the wall; and the interleaflet triangles, the regions of the wall that lie upstream (on the ventricular side) of the hinges, but are distal to the base of the sinuses that are found on the arterial side. Green = cardiomyocytes, blue = smooth muscle cells, yellow = fibrous tissue, purple = valve leaflet. The dotted line represents the position of the cross section through the valve complex. (**B**) Wholemount view of the adult mouse aortic root. The leaflets have been removed to allow the view of the other structures of the valve complex. (**C**,**D**) Masson’s trichrome images of the mouse aortic root at P21 in longitudinal and transverse planes. Red staining is muscle tissue, blue staining is fibrous tissue. c = commissure, h = hinge, ilt = interleaflet triangle, l = leaflet, s = sinus, sw = sinus wall.

The aortic and pulmonary valves, which sit within the arterial roots between the myocardium of the left and right ventricular outflow tracts and the aorta and pulmonary trunk, respectively, are structurally similar to one another, and are closely comparable in humans and mice [1,2,3]. Thus, in the mature heart both the aortic and pulmonary valves have three superficially similar leaflets that form the moving parts of the valve. The right (R) and left (L) sinuses of the aortic valve each host the ostium of a coronary artery, whilst the third non coronary/non-facing/posterior (N) and the three pulmonary valve sinuses are almost never associated with a coronary artery—even in congenitally malformed hearts. However, we now recognise the valve complexes to include more than just the three moving leaflets, with additional components including: the hinges, the attachment points of the leaflets to the wall; the commissures, the points of apposition of the leaflets close to the wall; the sinuses, pockets that form between the leaflets and the wall; and the interleaflet triangles, the regions of the wall that lie upstream (on the ventricular side) of the hinges, but are distal to the base of the sinuses that are found on the arterial side (Figure 1). The nomenclature used to describe the arterial roots is controversial. For example, the outflow tract has been divided by some authors into a proximal conus and a distal truncus, although this terminology is falling out of practice and three rather than two components are increasingly recognised. In this nomenclature, the distal component represents the intra-pericardial arterial trunks and the proximal component comprises the ventricular outflow tracts, with the valve complex appearing in the intermediate part [2]. In other contexts, anatomically artificial “rings” are described for clinically important measurements, although some publications have highlighted the misuse of these descriptions for developmental and anatomical purposes [4,5]. Thus, the development of this highly complex area remains the topic of speculation and controversy. One longstanding conundrum is, what are the similarities and differences between arterial and atrioventricular valve development, particularly those that relate to their pathology? How does the valve achieve an undulating crown-like attachment, crossing the arterial–myocardial boundary? How do the outflow cushions remodel to form the sculpted valve leaflets? Overall, the question of how aortic valve malformations relate to other cardiovascular malformations, both congenital and apparently acquired, has not been answered. The answer to all these puzzles may lie with the progenitor cells that form the arterial roots. Thus, a re-evaluation of arterial valve development focusing on the second heart field (SHF) and neural crest cells (NCC) may explain the relationships between these different elements and make sense of abnormalities in transgenic animal models and human malformations.

Cre-lox-based lineage tracing technology in the developing mouse has indicated how cells originating in the SHF and NCC are involved in the development of the outflow wall, as well as septal and valve structures. It is clear that the hinges and the leaflets have contributions from both SHF-derived cells and NCC [3,6]. Similarly, above the undulating leaflet hinge, the sinus wall is made up of vascular smooth muscle cells (SMC) that also derive from NCC and SHF. Within the roots, SHF-derived cells predominate, whilst more distally the entire tunica media is of NCC lineage. In the transition, these progenitors maintain separation as a thinning inner layer of NCC-derived SMC, and a thickening outer layer of SHF-derived SMC [3,7,8]. Proximal to the root, the myocardium is also derived from the SHF. Thus, the supporting structures for the leaflets, the hinge attachments and the interleaflet triangles, the fibroblasts and SMC at the base of the sinus and the subaortic and sub pulmonary myocardium, all arise from SHF progenitors and NCC to a greater or lesser extent. The question of the undulating attachment of the leaflets must be expanded to ask, how do the SHF-derived cells on the arterial side of the hinge become SMC whilst those on the ventricular side become myocardial? Moreover, how do the interleaflet triangles that are found on the ventricular side of the hinge attachments become fibrous, whilst apparently originating from the same SHF progenitors as the SMC within the sinus? Although these questions remain unresolved, it seems likely that local signalling events, rather than patterning within primitive fields of progenitors, are responsible for defining the precise characteristics of specific structures and tissues within the arterial roots.

## 2. Positioning of the Arterial Valves

The left and right leaflets of the aortic and pulmonary valves, as well as the septal leaflets of the mitral and tricuspid valves, are known to be derived from endocardial cushions. The main aspects of initial formation and positioning are similar for all these cushions, particularly those relating to cellular invasion through the endocardial to mesenchymal transition (EndMT) [9,10,11]. These endocardial cushions initially appear as expansions of pre-existing extracellular matrix (ECM; cardiac jelly) between the outer myocardium and inner endocardial layer, at approximately embryonic day (E) 9.5 in the mouse embryo (Carnegie stage (CS) 10–11 in the human embryo). In both the outflow tract and atrioventricular canal, the initial positioning of the cushions is regulated by local Bmp signalling and a complex interaction between several T-box transcription factors and Notch signalling (reviewed recently [11]). The expanded cardiac jelly of the outflow cushions is also in continuity with the much thinner layer of ECM that extends proximally towards the ventricular chambers. This can, at least in theory, allow for the movement of the cellular component of the forming cushions relative to the overlying myocardial wall. This slippage may allow for repositioning of the three valve primordia in each arterial root relative to each other and could also provide an explanation of how the undulating line of hinge attachment can later span both myocardial and arterial aspects of the roots.

**Figure 2:**
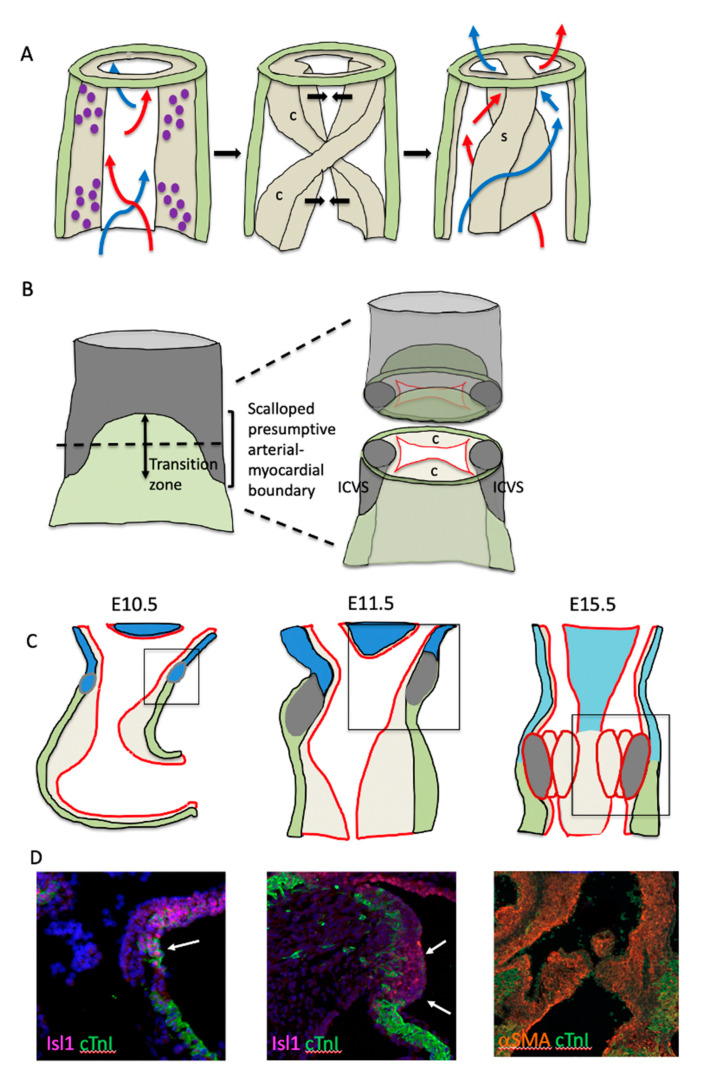
Early outflow tract development. (**A**) Model representing how flow of blood through the unseptated outflow tract may lead to formation of the spiralling outflow septum. Vorticial flow of blood through the outflow tract as the cushions are beginning to cellularise leads to aggregation of these cells (purple dots) in columns that prefigure the spiralling cushions (c). This positions and/or stabilises the cellularising cushions within the circumference of the outflow tract. The ensuing fusion of these cushions leads to formation of a spiralling septum (s) that places the root of the aorta to the left of the pulmonary trunk. (**B**) The boundary between myocardial and non-myocardial fate is first apparent at 10.5 in the mouse as the transition zone. The cells in this region are transitioning from a progenitor (grey) to a cardiomyocyte (green) fate. However, not all the undifferentiated SHF cells within this boundary region become cardiomyocytes. Two tongues of undifferentiated SHF cells can be seen to extend laterally into the myocardial outflow tract and these form the intercalated valve swellings (ICVS) that are the primordia of the posterior (non-coronary) and anterior intercalated leaflets of the aortic and pulmonary valves. (**C**,**D**) The myocardial-non-myocardial boundary is labelled by Isl1 (pink) and cTnI (green) at E10.5, with the cells in the transition zone labelled by both markers (arrow). By E11.5 the ICVS can clearly be seen in the outflow wall, as it continues to express Isl1 (arrows). By E15.5 the myocardial (green)-SMC (red) boundary at the level of the valve complex is well defined.

Whilst Bmp signalling determines the positioning of the forming cushions within the myocardial part of the outflow tract, little is known about how the original circumferential deposition of ECM becomes two longitudinal cushions in the superior–inferior opposition. Our previous studies have suggested that the aggregation of NCC within the outflow tract cushions affects where the discrete outflow cushions will form in the initially circumferential cardiac jelly [12]. It is possible that this localised NCC aggregation could be directed by signals from the surrounding myocardium or endothelium, however, it seems more likely that this is dictated by the physical properties of vorticial blood flow through the looping, but as yet unseptated, heart tube. Indeed, it has been shown that shear stress is asymmetrically localised in the outflow wall as the cushions are beginning to cellularise [13]; this could help to position and/or stabilise the cellularising cushions within the circumference of the outflow tract ([12]; Figure 2A). Later, the fusion of these spiralling cushions leads to placing of the root of the aorta to the left of the pulmonary trunk. The deletion of the heparan sulphate proteoglycan perlecan is associated with hyperplastic and malpositioned outflow cushions that ultimately result in transposition of the great arteries in a high proportion of mutant embryos [14]. In this model, the excessive numbers of mesenchymal cells (probably of NCC origin) do not form well defined “ridges” within the cardiac jelly. It can be imagined that this failure to form discrete aggregates of cells within the circumferential cardiac jelly will lead to aberrant positioning of the aorta and pulmonary trunk after septation. 

## 3. The Transition Zone and the Arterial-Myocardial Boundary

Initially, the main outflow cushions are completely contained within the SHF-derived myocardial part of the outflow tract [15]. However, at E10.5, SHF cells continue to add to the outflow tract and, although they initially remain undifferentiated, will later form SMC [3,15,16]. This produces a histologically defined arterial–myocardial junction within the aortic root. Thus, the defining process in the formation and positioning of the aortic root may be the switch from differentiation of the SHF progenitors to cardiomyocytes proximal to the boundary, to SMC distal to the boundary (Figure 2B–D). This boundary between myocardial and non-myocardial fate is first apparent at E10.5 in mice, as a region we named the transition zone [17]. The transition zone does not have an anatomical identity but is identified as a region with cells that express the SHF-specific transcription factor Isl1, but at the same time co-express differentiated cardiomyocyte markers. Thus, cells in this region are transitioning from a progenitor to a cardiomyocyte fate. Above the transition zone, the SHF progenitors are in continuity with similar cells in the dorsal pericardial wall and will later downregulate Isl1 expression; the majority of these will go on to form the arterial SMC of the aorta and pulmonary trunk. However, not all the undifferentiated SHF cells within this boundary region become cardiomyocytes. At E10.5 (CS12 or around 28 dpc in human), two tongues of undifferentiated SHF cells can be seen to extend laterally into the otherwise myocardial outflow tract ([15,18]; Figure 2B). By E11.5, these non-myocardial extensions, surrounded laterally by cardiomyocytes, form the intercalated valve swellings (ICVS; sometimes erroneously called the intercalated cushions—see later) and are the primordia of the posterior (P) aortic (non-coronary) and anterior (A) pulmonary intercalated leaflets [1,19,20]. The origin and remodelling of these ICVS to valve leaflets will be discussed in more detail later, but it is important to emphasise that their localisation within the wall, at the boundary between the myocardial and presumptive-arterial parts of the outflow tract, is unlikely to be coincidental to the remodelling of the main outflow cushions and may play an important role in defining the position of the arterial valve complex.

## 4. EndMT and Cushion Formation

The process of endocardial cushion mesenchyme formation has been reviewed extensively [10,11,21,22,23]. There is evidence from numerous sources that the outflow endocardium is derived from the second heart field (SHF). Thus, in lineage tracing studies utilising the “SHF-specific” *Isl1-Cre* and *Mef2c-AHF-Cre* mouse lines, outflow cushion endocardium and mesenchyme is labelled [24,25]. This contrasts with the atrioventricular endocardium where only a few cells are labelled by these SHF-specific Cre lines [6]. It remains to be seen whether there are significant implications of this different origin or whether, in practice, origin is of less importance than specification to undergo EndMT. EndMT initially occurs in the proximal outflow cushions, with only limited EndMT-derived mesenchyme observed within the distal cushions until after E11.5 [26,27]. Thus, EndMT-derived cells are not initially found in the region of the outflow cushions where the valves will form. The endocardium overlying the valve primordia expresses *Nfatc1*, which is important for valve development [28,29,30]. There appears to be two roles for *Nfatc1* in valve formation, playing an early role in initiating EndMT and then a later role in valve remodelling [30]. Cells labelled by *Nfatc1-Cre* (in which the *Nfatc1* has ever been active) remain in the endocardium and do not undergo EndMT [27]. Targeted deletion of *Nfatc1* in the endocardium results in an over-abundance of EndMT-derived cushion mesenchyme in the proximal outflow tract cushions at E10.5, and an extension of these cells into the distal region [27], perhaps indicating that EndMT continues unchecked in the absence of *Nfatc1*. The observation that the arterial valve leaflets later appear under-developed in the mutant embryos [28,29,30] seems counter-intuitive, but it may be that appropriate interactions between EndMT-derived cells and NCC are essential for leaflet remodelling, and that if this balance is disrupted, underdeveloped leaflets result. Many other signals that induce or repress EndMT of endocardial cells have now been described, including the Notch, BMP and TGFβ signalling pathways [31,32,33,34]. For example, the Nf1/Ras pathway has also been implicated in repressing EndMT in the outflow and atrioventricular cushions [35,36,37], with exacerbated Ras signalling leading to hyperplastic cushions. 

**Figure 3:**
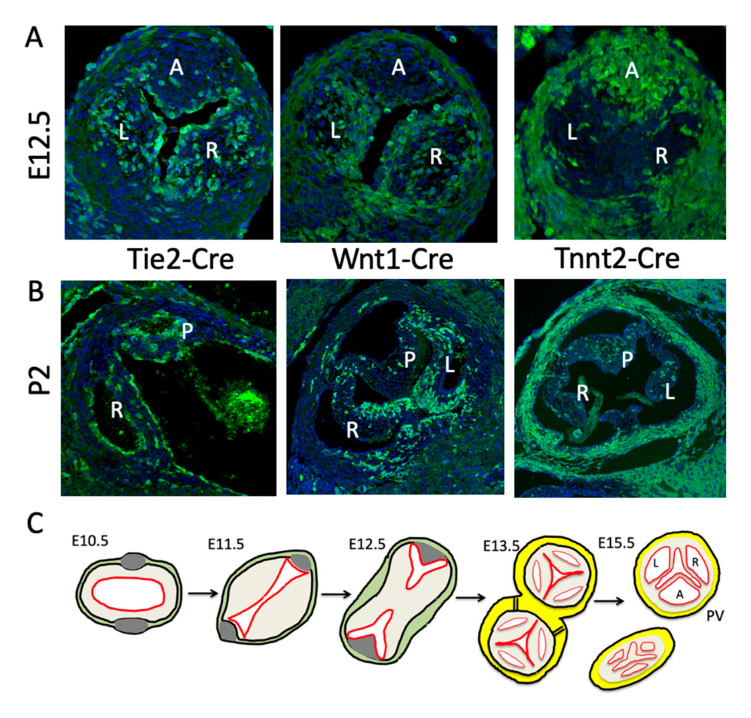
Lineage tracing of cells in the foetal and neonatal arterial valves. (**A**) At E12.5, *Tie2-Cre* labelled endocardial-derived cells, *Wnt1-Cre*-labelled NCC and *Tnnt2-Cre* labelled cells derived directly from the SHF, via the outflow wall, are found within the pulmonary valve leaflet primordia. Whilst *Tie2-Cre* and *Wnt1-Cre*-labelled cells are most abundant in the left (L) and right (R) leaflets, the *Tnnt2-Cre* labelled cells are the most abundant in the anterior (A) leaflet derived from the ICVS. Similar observations are made for the aortic valve (not shown). (**B**) At P2, each of the three lineages of cells are maintained within the leaflets of the aortic valve. As at E12.5, *Wnt1-Cre* and *Tie2-Cre* labelled cells are most abundant in the L and R leaflets, whilst *Tnnt2-Cre* labelled cells are most abundant in the posterior (P) leaflet derived from the ICVS. Similar observations are made for the pulmonary valve (not shown). (**C**) Cartoon illustrating how arterial valve leaflet formation is intimately linked to outflow tract septation. At E10.5, the forming endocardial cushions (buff coloured) are circumferential within the outflow vessel and the ICVS (grey) can first be seen within the outflow wall (green). By E11.5 the endocardial cushions (buff) have expanded and are coming together to fuse in the midline. Fusion of these cushions separates the outflow tract and gives rise to the L and R valve primordia. The ICVS give rise to the A and P valve primordia. By E13.5 the aortic and pulmonary valve are distinct, and the sinuses are beginning to appear. At E15.5, valve sculpting is almost complete, and the aorta and pulmonary valves are offset. Green = myocardium, yellow = SMC, red = endocardium.

Although EndMT-derived cells are not initially found in the distal outflow cushions, by E12.5 they are abundant in the forming leaflets [12,26,38] and are maintained in the postnatal valves (Figure 3A,B). These endocardial-derived cells (EDC) seem to play a crucial role in regulating the growth and remodelling of the cushion. There seems to be a functional difference between early endocardium and the *Nfatc1*-expressing late valve endocardium regarding Notch signalling. The loss of Notch signalling from the endocardium before EndMT results in grossly hypoplastic cushions and early death. However, if this stage is bypassed, perhaps by using *Nfatc1-enCre* (valve endocardium-specific [27]) rather than *Tie2-Cre* (all endocardium and endothelium [39]), or by deleting a Notch ligand that is only active in the endocardium after EndMT (e.g., [40]), then thickened arterial valve leaflets are the usual result. Recently, it has been suggested that some macrophages in the late foetal and postnatal valve mesenchyme are derivatives of the endocardium [41]. However, it remains unclear whether other EndMT-derived cells in the interstitium play specific roles within the adult valve.

## 5. Neural Crest Cells, Outflow Tract Septation and Valve Development

Outflow tract septation is intimately involved in arterial valve formation through shared cells, cushions and timing of events. Analysing human embryos, Kramer [19] showed that the fusion of the main outflow cushions (at around CS 12–13 or ~30 dpc in human embryos, corresponding to E11.5 in the mouse embryo) produces the precursors of the right and left leaflets of the forming arterial valves, with the parietal main outflow cushion contributing to the left leaflets of the aortic and pulmonary valve, and the septal leaflet contributing to the right leaflets (see Figure 3C). The fusion of the main cushions separates the channels of the aorta and pulmonary trunk and subsequent separation of the aorta from the pulmonary trunk in a plane perpendicular to the line of cushion fusion, results in the formation of two, free-standing arterial trunks with their distinct valves. This corresponds well with what is seen in the mouse embryo (Figure 3; [12]). This septation is dependent on NCC and cannot occur if there are significantly reduced numbers within the outflow cushions [42]. 

A great deal is known about NCC and their developmental roles outside the heart. NCC are a population of progenitor cells that arise in the dorsal neural tube and migrate throughout the body to contribute to a variety of cell types and organ systems, including the heart [43]. There is an extensive literature reviewing the processes of NCC induction, migration and differentiation (for example [44,45,46]) and these will not be considered further here. In contrast, although there have been many studies that have shown that NCC are critically required for outflow tract septation, their roles in heart development are relatively poorly understood and there have been only limited studies in recent years (reviewed in [47,48,49]). NCC migrate through the pharyngeal arches and first appear in the cardiac jelly of the distal outflow cushions early on E10 of mouse development (CS11 in human). At this stage, the distal outflow cushions are acellular, as although EndMT is initiating in the outflow cushions, this occurs in the proximal region. NCC that remain in the pharyngeal region rapidly differentiate into SMC and in doing so stabilise the nascent endocardial tubes that will become the pharyngeal arch arteries [50,51,52]. In contrast, the NCC entering the outflow cushions initially remain relatively undifferentiated (do not express for example αSMA or SM22α; [3]) and retain their expression of early NCC markers including *PlexinA2* and *Ap2α* [53,54]. The main function of these NCC appears to be to provide bulk to the distal cushions, causing them to come into sustained contact, and permitting fusion, with each other and the dorsal wall of the aortic sac [15]. This fusion event initiates outflow tract septation and separates the systemic (aortic) and pulmonary circulations. Once septation is complete, the majority of the NCC in the proximal cushions die by apoptosis [55] and are replaced by ingrowth of myocardial cells to form the muscular subpulmonary infundibulum [56,57]. It has been suggested that the NCC might be involved in defining the position of the arterial valves, as at E11.5, NCC are restricted to the distal cushions and appear to form a boundary with the EndMT-derived mesenchymal population in the proximal region [27]. However, this is a transient boundary, as by E12.5, NCC are found throughout the outflow cushions, apart from their most proximal tips [15,52]. Whilst the role of NCC in the proximal outflow tract appears to be a transient one, the NCC make a permanent contribution to the arterial valve complex, contributing to the fibroblast-like cells found within the commissures, interleaflet triangles and the leaflets of the valves (Figure 3A,B; [3]). How the NCC-derived cells in these different components of the valves differ from one another remains unclear, and they may have more similarities than differences. Unfortunately, there is no mechanism currently to specifically disrupt the NCC that contribute to the valve leaflets, leaving those that are involved in other structures within the arterial roots, or those required for septation, intact. Thus, we do not know for certain if loss of these cells, in the setting of normal outflow septation, would disrupt valve formation and/or remodelling.

## 6. Direct Differentiation of SHF Progenitors into Valve Mesenchyme

**Figure 4:**
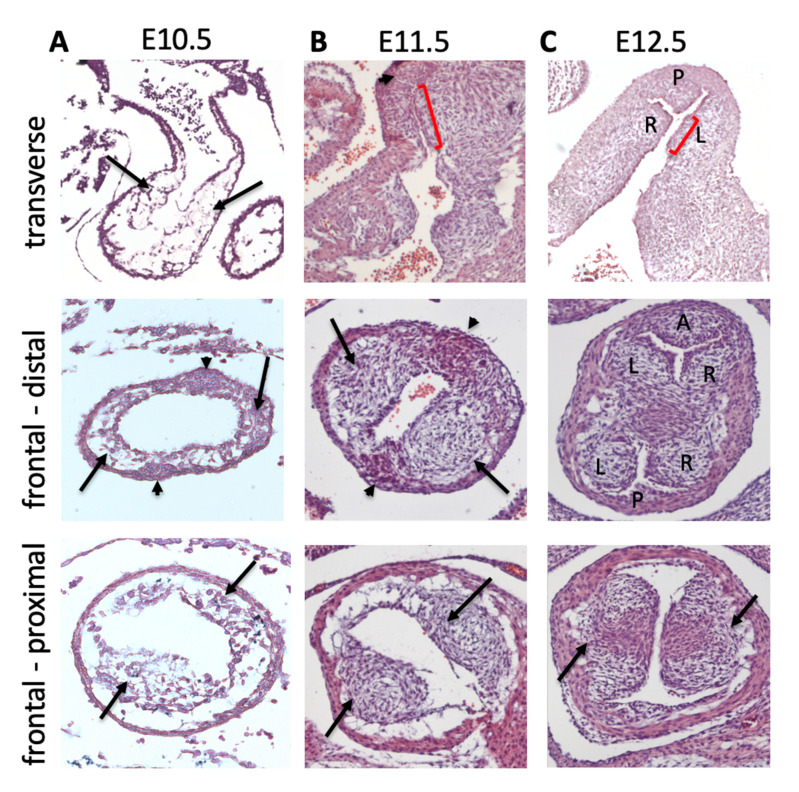
Contributions of the cushions and ICVS to the arterial valve primordia. Histological sections of mouse hearts stained with H&E. (**A**) At E10.5 the outflow is unseptated. The cushions (arrows) are forming and can be seen to extend along the length of the outflow tract in transverse view and to be circumferential in both the distal and proximal regions, in frontal view. The ICVS cannot be seen in transverse sections at this stage, but in frontal sections can be seen distally as cell dense regions within the outflow wall (arrowheads). (**B**) By E11.5, the cushions are more cell dense and in transverse sections the valve forming part (red bar) can be seen to be distinct from the proximal part of the cushions that will form the sub-pulmonary infundibulum in transverse sections. The ICVS (arrowhead) can now be seen adjacent to the valve-forming parts of the cushions. In frontal sections the main cushions (arrows) are coming together towards the midline of the outflow tract in the distal region. The ICVS (arrowheads) have expanded but are still continuous with the outflow wall. More proximally, the cushions (arrows) are still widely separated. (**C**) By E12.5 the outflow tract has septated and the primordia of all three leaflets are apparent in transverse and frontal views. The proximal region of the outflow tract remains unseptated at this time point, although the cushions (arrows) are beginning to come together. A = anterior leaflet; L=left leaflet; P = posterior leaflet; R = right leaflet.

The intercalated leaflet valve swellings (ICVS), the precursors of the anterior/posterior leaflets, were first noted by Kramer [19] and are seen clearly in cross sections through the outflow tract as lateral bulges within the outflow wall ([20]; Figure 3 and Figure 4). Importantly, although they are overlaid by a thin layer of ECM, the bulk of these structures is completely distinct from the main endocardial cushions and, at their first appearance at E10.5, they do not contain ECM or form by EndMT. Nor do they contain significant numbers of NCC [12,20,58,59]. Lineage tracing studies have shown that the majority of the cells contained within the ICVS are derived from the SHF [20,59], with much smaller contributions from EndMT or NCC than are seen for the main endocardial cushions [12,20,58,59]. Although in some ways they have similarities to the lateral cushions that form the mural leaflets of the mitral and tricuspid valve [20], they do not contain cells derived from the epicardium. Thus, they are distinct from other valve-forming structures within the developing heart. Although the ICVS are continuous with the outflow wall at E10.5–E11.5, they do not label with cardiomyocyte markers and are instead immuno-labelled with antibodies specific to Isl1. Whilst maintaining this progenitor-phenotype, they begin to express Sox9, a characteristic marker of cushion mesenchymal cells [60], and thus differentiate to this fate without passing through the endocardial lineage during EndMT. The conclusion from this is that the ICVS are not endocardial cushions and that the cells within them differentiate directly from the SHF [20]. The confusion arises because although these ICVS arise from the outflow wall and their early formation is entirely distinct from the main outflow cushions [20,58], they rapidly take on the superficial appearance of endocardial cushions by generating large amounts of ECM and expressing typical cushion markers such as Sox9 [20]. Hence, by E12.5 they are almost indistinguishable from other cellularised valve primordia originating from endocardial cushions. Thus, after septation it is reasonable to called them “intercalated cushions”. However, subtle differences remain, for example, the ICVS are more cell-dense and express tropoelastin which the primordia derived from the main cushions do not (Eley, Chaudhry, Henderson; unpublished data). As well as having distinct origins, the ICVS also appear more distally than the right and left cushions, although later remodelling places all the leaflets within each root on their own orthogonal planes. Despite these later similarities, the undifferentiated SHF cells that form the ICVS can still be recognised as they are labelled by the “myocardial-specific” *Tnnt2-Cre* transgenic marker ([20,58,61]; Figure 3A,B). Despite this they are not transdifferentiated cardiomyocytes (they never at any point express markers of differentiated cardiomyocytes) but at a very early stage activated the promoter used to produce the *Tnnt2-Cre* transgene. Whilst the *Tnnt2-Cre* line is therefore extremely useful for following the ICVS cells, it is important to recognise that if is used to drive Cre in the myocardium, there could also be direct effects on arterial valve development. 

Although the mechanisms underpinning the formation of the ICVS have only recently been elucidated, we do have some ideas about the molecular mechanisms that are likely to be involved as there are several mouse mutants where the non-coronary leaflet is missing or hypoplastic. This includes mice null for *Tbx1* [62], *Alk2* [63], *Robo1/2* [64] and when *Vangl2* is knocked out specifically in the developing SHF [20]. Of particular interest, Notch1-ICD and Jag1/2 are localised within the ICVS during its formation at E10.5–E11.5 [20], in a pattern that suggests that Notch signalling may play an important role during their development. Although it is clear that Notch signalling is important in valve development in general, there is also specific evidence that it is important in the developing ICVS. Knockout of *Jag1* and *Jag2* in the developing ICVS using *Tnnt2-Cre* results in defects in the formation of the ICVS that result in a dysplastic or missing non-coronary leaflet of the aortic valve (and to a lesser extent the posterior leaflet of the pulmonary valve; [20]). Thus, there appears to be a specific signalling network acting within the ICVS to bring about direct differentiation of SHF cells to valve mesenchyme; this network utilises Notch signalling. 

In summary, it seems that NCC and SHF-derived cells (both via direct differentiation and transitory passage though the endocardial state) are crucial to the formation of arterial valve primordia, although their roles in these processes may be complimentary rather than overlapping. Further work will be needed to better define these specific requirements and roles. 

## 7. Cushion Expansion to Form Arterial Valve Primordia

The period between E10.5 and E12.5 is crucial for the appearance of discrete arterial valve primordia that remodel at the distal end of the outflow cushions, distinct from the septal components that form from the proximal cushions (Figure 4). Although the factors that regulate this process remain unclear, the presence of the transitionary tissues at the myocardial–arterial boundary, or indeed the formation of the ICVS adjacent to the main cushions, may be significant. Thus, it is possible that unique factors within the boundary region influence the development of the adjacent cushion tissue to become valve-like, whilst the more distant proximal cushion acquires a septal phenotype, becoming muscularised by cardiomyocytes migrating from the enveloping myocardial outflow wall [56,57]. The resolution of this point will require further investigation. 

Once the relevant progenitor lineages have contributed cells to the mesenchyme by late on E10.5, they undergo a period of proliferation that results in rapid cushion growth. A number of molecules are implicated in this process, most of which affect both the atrioventricular and outflow regions. These include Vegf and FGFs (particularly FGF4) which are major drivers of cushion mesenchyme proliferation [65,66,67,68]. Other genes negatively regulate cushion mesenchyme proliferation, for example, EGF plays an important role in limiting proliferation in the cushion mesenchyme, with mice hypomorphic for the EGF receptor having increased mesenchymal cell numbers and enlarged arterial valve leaflets [69]. Similarly, Jag1, a Notch ligand, also appears to limit cushion mesenchyme proliferation [40]. Thus, too much [35,36,37,70], or too little (for example [71,72]) cell division is associated with defects in the leaflets as development proceeds.

As well as increasing cell numbers, the ECM matures and increases in complexity as the cushions expand. Knockout of the ECM components found in the remodelling cushions, such as versican, frequently result in acellular cushions (e.g., [73,74]) as these molecules are required for cushion formation as well as remodelling. Thus, it will take more nuanced approaches to reveal the specific roles for these molecules in the latter stages of cushion development and maturation. However, mouse embryos lacking Adamts5, which is required for versican cleavage, have grossly hyperplastic arterial valves (interestingly the pulmonary valves are most affected) due to the accumulation of uncleaved versican that, as well as increasing the ECM component, also results in increased mesenchymal cell numbers, with abnormalities seen as early as E12.5 [75]. Thus, along with its early role in supporting EndMT, versican also appears to be important for remodelling of the cushion/valve mesenchyme.

Recently, there has been much interest in the potential role of cilia in the development and maintenance of valve leaflets, largely because of the association between mutations in cilia-associated genes and valve defects (e.g., [76]). Primary cilia are small projections of membrane with a microtubule core that are implicated in cell signalling and mechano-sensation [77]. There is considerable evidence to show that the presence of cilia is temporally and spatially regulated during valve formation, and that although they are rarely found on the surface of endocardial cells, they are found on almost all cushion mesenchyme cells at mid gestation [78]. Cilia numbers decrease on the cushion mesenchyme from E11.5 onwards, declining to around 15% of adult VIC [78]. Although the reasons for this decline are not clear, it may be related to differentiation, as in a variety of cell types, cilia are required for this process [79]. The loss of cilia from the EndMT-derived component of the cushion mesenchyme (using *Nfatc1-Cre* to delete *Ift88*) resulted in dysplastic aortic valves, with an enlargement of the hinge region between the leaflets suggestive of BAV, and over-expression of ECM molecules including versican and collagen I [78]. Although it is unclear when or how the BAV arose, this suggests that cilia regulate ECM production or turnover in the developing valves and may play a similar role in the adult. This is supported by other studies that have shown that defects in cilia can lead to mitral valve prolapse, a disease where myxomatous degeneration is a characteristic feature [76]. It is not clear whether a specific subset of VIC retain a cilium in the adult valve, although it is interesting to speculate that these cells are the ones that are activated in response to stress. Absence from the valve endocardium suggests that their role is unlikely to be related to the detection of shear stress. 

## 8. Valve Sculpting 

**Figure 5:**
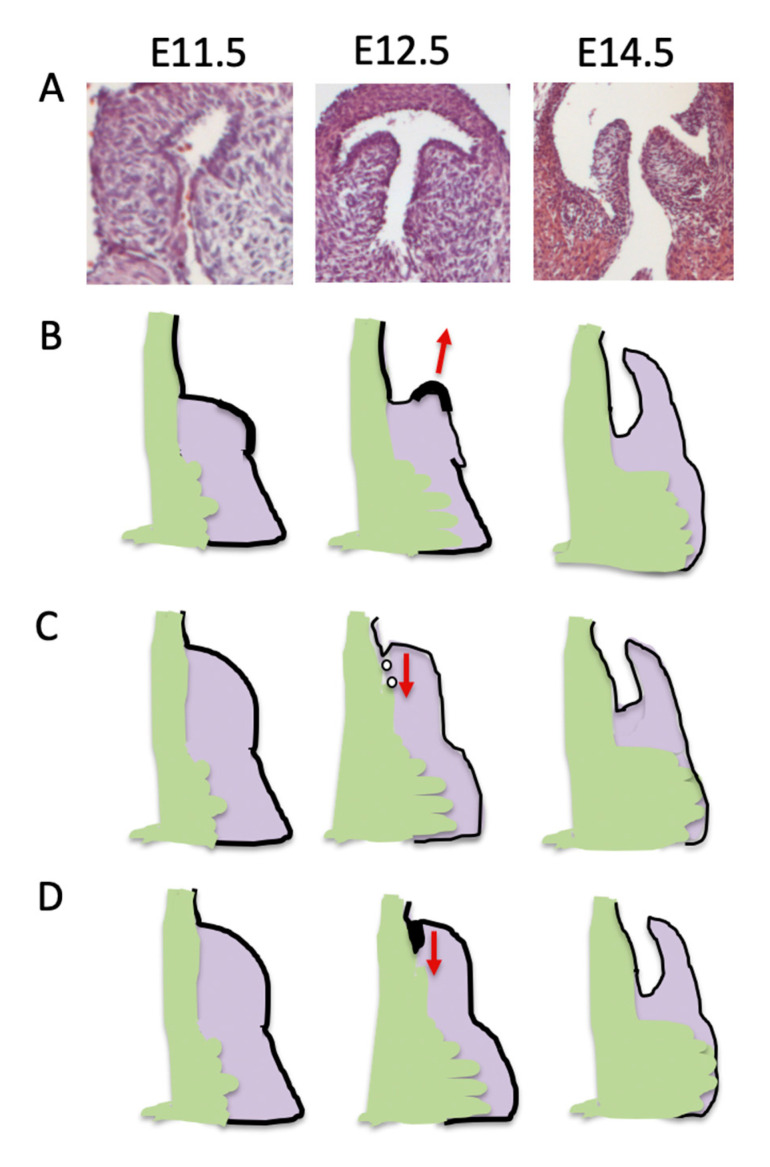
Models of valve sculpting. (**A**) Histological images showing how the leaflet sculpts from a bulky cushion at E11.5, to a well-defined leaflet by E14.5. (**B**) The leaflet elongation model proposes that under the influence of factors secreted by the thickened valve endocardium, the leaflet actively elongates to acquire its sculpted form. (**C**) In the second model a process of cell death removes cushion tissue at the boundary with the outflow wall and in this way excavates the sinus, leading to freeing of the leaflet. (**D**) In the final model, the endocardium bends into the cushion tissue and in so doing creates the sinus, again freeing the leaflet. Experimental evidence is minimal for each of the three models and further work is needed to determine which, if any, are accurate.

Over the remaining days of foetal and early postnatal life, the bulky, primitive valve precursors are sculpted into thin fibrous leaflets, connected to the vessel wall by hinges that delineate the sinuses (Figure 1). These sculpting stages of arterial valve development remain the least well understood but may be the most relevant to human disease. Recent studies generally refer to a process of valve elongation or lengthening that is required to produce well-functioning and coapted leaflets [11,80,81,82]. However, by E11.5 in the mouse heart, before the outflow tract has septated and the cushions have remodelled to form distinct valve primordia, the outflow cushions cyclically occlude the outflow tract [83], allowing only unidirectional flow of blood. Thus, the valve leaflets do not need to lengthen in order to coapt (and thus function efficiently) when the valve is closed. This suggests that lengthening of valves may not be necessary, but that maintaining the overall proportions of valves during growth and enlargement of this area may be more important. The molecular mechanism(s) underpinning sculpting of the arterial valve leaflets have not been described in detail although there are some hypotheses, with varying amounts of supporting data. One hypothesis suggests that cushion mesenchyme is removed close to the wall, by a process of cell death. High levels of apoptotic cell death have been reported in the developing outflow cushions [84,85,86,87,88] and removal of cushion tissue by cell death in order to refine their shape continues to be an attractive model or leaflet sculpting (Figure 5). However, apoptosis has not been described in the sculpting arterial valve leaflets, although detailed studies are lacking. In contrast to cell death, phagocytosis (cell engulfment), carried out by macrophages derived from the endocardium, plays an essential role in remodelling the mature leaflets and in their absence, the leaflets become dysplastic [41]. Thus, the targeted removal of cushion mesenchyme and/or turnover of mature interstitial cells may be an important component of leaflet sculpting and homeostasis. Alternatives mechanisms of leaflet sculpting have been suggested. Early investigations, before the era of molecular biology, utilised electron microscopy to show that there is ingrowth of a thickened endocardium into the outflow cushion mesenchyme in both chicken and mouse embryos (from approximately E12.5; [89,90]). Furthermore, it was suggested that this ingrowth is associated with cell death of endocardial cells, although the data to support this was minimal. The valve endocardium expresses a number of signalling molecules including Fgfs, Wnts and Hh [68,91,92] and similarities have been drawn with the apical ectodermal ridge that acts as a signalling centre for limb bud outgrowth [22]. These signalling molecules may modify proliferation, differentiation and/or cell death within the underlying cushion mesenchyme, thereby regulating the process of valve leaflet formation and sculpting. Although there have been no detailed studies of how the valve sinuses form since those of Hurle, 40 years ago [89,90], it seems possible that their formation is intimately linked with leaflet sculpting, as the cavity of the sinus takes up much of the space previously occupied by cushion tissue (Figure 5). Whatever the cellular mechanism, high resolution episcopic microscopy (HREM) has revealed that the first indications of cushion excavation to form the sinuses are apparent late on E12.5 of mouse development [2], and that the part of the valve primordium closest to the muscular walls of the arterial root excavates, whilst the part closest to the lumen is retained as the valve leaflet. As the gradual excavation of the sinus simultaneously results in the free (lumenal) edge of the valve leaflet getting longer, this may explain why the valve has been interpreted as lengthening as it remodels (Figure 5). 

Although the precise mechanisms are unclear, evidence from the zebrafish and chicken embryo suggest that haemodynamic forces play an important part in the morphogenesis [93] and remodelling of cushions to form sculpted valve leaflets [94,95,96,97]. In the absence of normal blood flow through the heart, either as a result of reduced contractility, disturbed flow resulting from anatomical alterations, or the absence of molecules that sense the flow and send signals that lead to a change in cell/tissue behaviour, the cushions remain bulky, are malpositoned and the leaflets do not remodel properly. Although a detailed discussion of this topic is beyond the scope of this manuscript, there have been several excellent reviews published recently (for example, [98,99]). These have highlighted the importance of shear stress in sculpting the leaflets and have shown that mechanosensors in the valve endocardium activate the transcription *Klf2*, which brings about the cellular changes involved in valve sculpting via a Wnt signalling cascade [94,100]. For example, Piezo1, a mechanosensor, has been implicated in valve sculpting [101]. In the absence of this mechanosensory signalling cascade, the cushions fail to remodel appropriately resulting in dysplastic leaflets. It should be noted that as the mechanisms underpinning valve sculpting remain unclear, the details of how shear stress brings about this remodelling are correspondingly poorly defined.

## 9. Maturation of the Arterial Valve Leaflets

Mature valve leaflets contain valvular interstitial cells (VIC) that are derivatives of the cushion mesenchyme. These VIC form three cellular layers, characterized by distinct ECM profiles: the fibrosa (on the arterial side of the leaflet) composed mostly of collagens; the spongiosa made up mostly of proteoglycans; and the ventricularis, which contain elastin fibres. The leaflets are also covered by a specialised endocardial layer (valve endocardial cells; VEC) [80]. This stratification of the valve into its three layers occurs relatively late, first becoming apparent at approximately E16.5 of mouse development [80], at the end of the first trimester in human embryos. Initially, collagens and proteoglycans, including versican, hyaluronan and perlecan [14,102,103] are widespread in the developing cushions. Elastin, in contrast, is found only at low levels in the valve leaflets before birth in the mouse heart (Henderson; unpublished data), although it has been shown to be present by Hamburger and Hamilton stage 30 in the chicken embryo ([104]; equivalent to approximately E13.5 in the mouse) and as early as 7 weeks of gestation in developing human arterial valves [105]. However, after birth, elastin is markedly upregulated in mammalian valves and it is well recognized that remodelling of the ECM continues into postnatal life [80]. Recently, the relationship between the three layers of extracellular proteins and subgroups of VIC has begun to be elucidated [106]. Thus, subpopulations of VIC that express high levels of fibrillar collagen, as well as other genes known to be involved in fibril organisation and ECM maturation have been identified at postnatal day 7 in the mouse heart. Similarly, VIC expressing high levels of glycosaminoglycan-containing proteins including versican, fibulin-2 and lumican, have been identified at the same stage [106]. Surprisingly, elastin was expressed by both subgroups, but a specific elastin-rich subgroup of VIC was not identified. Indeed, there is a disconnection between the initial lineage of VIC and those in mature valves. Whilst mouse studies have shown that the distinct embryonic lineages that give rise to VIC are differentially localised within the foetal and postnatal valves [3,12,107], this has not yet been mapped in the adult valve and it remains unknown how the subgroups of VIC identified on the basis of their transcriptome [106] correspond to the different lineages, or to different disease states. It is possible that distinct embryonic lineages may predispose to disease and this is supported by the observation that when only one of the three leaflets is thickened, it is usually the non-coronary [108], suggesting that its different origin may predispose it to disease in some scenarios. However, studies where the three valve leaflets are examined separately are rare. Current evidence leads us to believe that there are distinct groups of VIC (potentially with distinct origins) with differing roles and susceptibilities to disease, but there may be a significant degree of overlap between these factors. Whilst all three lineages (EndMT-derived, NCC and directly differentiated SHF-derived cells) are retained into postnatal life [12,20,39,43,109], cells derived from the bone marrow—largely macrophages and dendritic cells—are mostly found in the valve leaflets postnatally, expanding to make up almost 20% of cells in the valve mesenchyme by a few weeks after birth [110,111,112]. Other studies have suggested that some macrophages in the valve interstitium are derivatives of the endocardium and that these cells appear to be more phagocytic than those derived from the bone marrow [41]. In healthy, mature valve leaflets, the VIC are generally quiescent with only a low level of cell turnover. These cells maintain normal valve structure and function and express a variety of markers that place them within the phenotypic spectrum of fibroblasts (although they are distinct from fibroblasts in other tissues). Similarly, the endocardial and immune cells found in the arterial valves appear to change little in the first few weeks of postnatal life. However, in response to disease or injury, a subpopulation of these apparently quiescent VIC can be activated to take on features of myofibroblasts. This activation occurs in response to signalling factors such as TGFβ which rapidly results in the formation of stress fibres and alignment, leading to increased contractility and increased mechanical stress. This may be the first step in a pathway that ultimately leads to gross remodelling of the valve matrix and valve disease. In diseased valves, some VIC begin to differentiate into osteoblasts to promote calcification of the leaflets. The transcriptional and signalling factors underlying this transition have been reviewed in detail [80,113]. However, the lineage of these VIC has not yet been determined. It is equally possible that different lineages have specific functions, or conversely, that there are no functional differences between them, with each are as likely as any other to remain in the mature valves.

## 10. Mechanisms Underpinning BAV

Congenital malformations of arterial valves include valve leaflet dysplasia, in which the leaflets are typically thickened and/or shortened, and valves with abnormal numbers of leaflets, including unicuspid, bicuspid and quadricuspid variants [114,115]. In all cases, the valves are predisposed to stenosis and/or regurgitation and degeneration in later life. Clinical series from the U.S. and Europe have suggested a common left-right leaflet (LR) occurs in about 85% of non-syndromic BAV cases [116,117]. However, studies from South Korea and Japan suggest that the LR pattern may be found in less than 60% of Asian patients [118,119]. Common left/non-coronary (LN) leaflets are rare in any population. Recent studies have suggested that genetic syndromes can be associated with different patterns of leaflet fusion. For example, Down’s syndrome is associated with a common RN leaflet, whilst Turner and DiGeorge syndrome with a common LR leaflet [120]. Together, all this evidence suggests that there is likely to be a strong genetic element to leaflet patterning. A well-recognised feature of bicuspid valves is the presence or absence of a raphe, presumed to represent a fusion seam, which in some cases is associated with a notch or an asymmetry suggesting a third leaflet formed during development, but fused with one of the others. Although it is commonly stated that 85–90% of BAV have a raphe [116,117], recent studies suggest as many as 25% may have no raphe [121].

**Figure 6:**
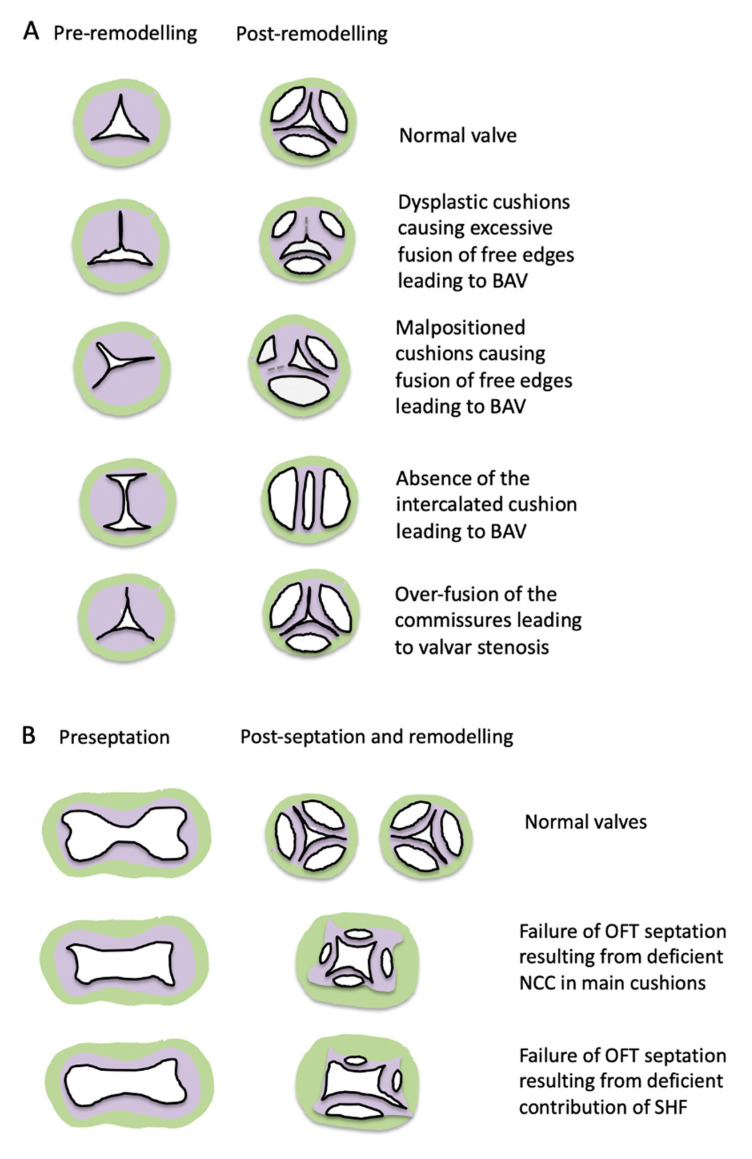
Possible mechanisms underlying THE bicuspid aortic valve BAV. (**A**) Cartoon illustrating several possible mechanisms underlying BAV in the situation of outflow tract septation. These can result in different numbers of sinuses and raphes and can occur early in development (as shown) or later in development as a consequence of pathological processes. Valvar stenosis could also be caused by over fusion of the cushions, at the commissures. (**B**) Cartoon illustrating possible valve outcomes when outflow septation fails. In the case of the proposed situation where the SHF is deficient, the numbers of leaflets can be highly variable, possibly depending on the extent of deficiency.

Numerous mechanisms to explain development of BAV have been proposed (Figure 6A) over the last century [122,123,124,125,126,127]. These include excessive cushion/leaflet fusion, failure to form the main cushions, abnormal outflow tract septation, and the absence of one of the leaflet primordia, primarily the ICVS that will form the non-coronary leaflet. Although many of these suggestions were made decades before developmental molecular genetics existed, these potential mechanisms remain and there is evidence for each of them as potential mechanisms for BAV in the literature today. 

## 11. Hyperplastic Cushions/Leaflets and Excessive Fusion

Whilst we now know that severe disruption of EndMT results in acellular or severely hypoplastic cushions and embryonic death in mid development (e.g., [128]), it remains possible that more subtle defects, for instance those that prevent appropriate resolution of EndMT, might disrupt cushion positioning or lead to hyperplastic cushions and leaflets that are forced into apposition and thus are more likely to fuse. There is accumulating evidence to support the idea that cushion or leaflet fusion may be a cause of BAV. BAV resulting from excessive fusion of the main outflow cushions has been described in the Syrian hamster, leading to LR fusions. This includes BAV and more rarely bicuspid pulmonary valves (BPV; [126,129,130]) as well as quadricuspid leaflets, all in the same colony of animals [130,131]. An investigation of the developmental defects underlying the abnormal leaflet fusion suggest that hyperplasia of the main cushions shortly after outflow septation pushes the cushions into apposition and this leads to fusion; a fusion seam can sometimes but not always be seen later in gestation in these animals [126,132]. Interestingly, in an inbred colony of the hamsters, even those animals determined to have a tricuspid valve frequently had excessive fusion of the left and right leaflets [132] suggesting that the affected gene (currently unknown) shows variable expressivity, or that other non-genetic factors affect the phenotype. Quadricuspid aortic valves in the outbred colony appear to result from splitting of one of the cushions (usually the right) into two relatively undeveloped cushions [133]; how this relates to the BAV remains unclear. 

Recently, another well-described example of leaflet fusion, but this time later in gestation, has been described. In this case *Krox20*, a transcriptional regulator, was analysed during heart development revealing that the loss of *Krox20* in NCC (and more rarely when removed from EDC) can lead to BAV [134,135]. Interestingly, the loss of *Krox20* increased the numbers of NCC whilst the numbers of the EDC population remained normal. Excessive numbers of NCC, particularly in the non-coronary leaflet, led to malpositioned and hyperplastic leaflets that fused by late gestation [135]; a raphe could clearly be seen in these cases of BAV. These studies thus confirm that fusions between the cushions or leaflets, at different stages of development, can lead to BAV.

## 12. Displaced Cushions

A novel mechanism for the development of BAV has been suggested based on anatomical examination of a series of human embryos [136]. These authors observed that in some embryos the aortic ICVS was displaced proximally, with the developing valve tissue first appearing external to the myocardium. The valve then appeared to develop without this displaced ICVS, giving rise to a biscuspid phenotype. Interestingly, this did not appear to occur in the developing pulmonary valve, suggesting that there may be some subtle differences between the formation of the aortic and pulmonary valves [20,136]. The abnormal positioning of the outflow cushions in early development has been suggested to cause BAV in *eNOS* mutants [137]. In this case, the endocardial cushions were found to be misplaced and the main cushions were fused with the ICVS. More recently, this has been suggested to result from relatively small changes in the contributions of SHF and NCC to the forming cushions [59]. These authors also suggested that the BAV in the eNOS null mice might arise from a distinct mechanism, where the precursor of the anterior/posterior leaflet fails to separate from the main cushions. However, this implies that the ICVS does not exist as a separate entity, and thus is at odds to what we have described earlier, and what seems to be the generally accepted view [2,12,18,19,20,58]. Interestingly, it has been shown that *Krox20* can regulate *eNOS* expression, and that mice heterozygous for mutations in *Krox20* and *eNOS* develop BAV, whereas single heterozygous mutants do not [138], supporting the genetic interaction between these two genes. Similarly, abnormal cushion positioning in mice expressing a dominant-negative form of Rho kinase (ROCK; a modifier of the cytoskeleton) in the NCC lineage results in their fusion. In this case, the abnormal positioning of the cushions appears to result from disrupted aggregation of NCC within the forming cushions [12]. 

## 13. Bicuspid Valve without Raphe and the Absent Leaflet

Although the majority of human BAV present with a raphe [115,116] some studies suggest that BAV without raphe is more common than generally reported. For example, Koenraadt et al. [120] suggested that absence of raphe was represented in 25% of Dutch patients with BAV. Moreover, in cases of BPV, the majority also had BAV without raphe, suggesting that whatever process causes bicuspid valves without raphe, it affects both valves at the same time [139]. However, it should be remembered that a raphe might be present initially and then disappear later, particularly if the fusion event occurred very early in development [125]. When the complete absence of a leaflet is considered, the non-coronary leaflet is usually suggested to be the missing one [20,62,63,140]. As the non-coronary leaflet has a distinct origin from the main cushions (although the SHF is crucial to all; [20,59]) it can readily be seen how formation of this leaflet could be disrupted whilst leaving the right and left leaflets intact and with no evidence of raphe. Interestingly, there appears to be an over-representation of this type of BAV (absent non-coronary leaflet) within the mouse literature, suggesting that this mechanism may be common, at least in this species. 

## 14. Abnormal Leaflet Numbers in the Setting of Defects in Outflow Tract Septation

Abnormalities in outflow tract septation, for example, common arterial trunk, are accompanied by defects in the arterial valves, with dysplasia and abnormal numbers of leaflets seen in the common truncal valve. However, the number of leaflets observed varies, with 3 or 4 leaflets being most common. It seems probable that the number of leaflets varies according to the cause of the common trunk, with abnormalities in cushion fusion, as is seen in the setting of NCC deficiency, usually resulting in four leaflets, (for example the *Splotch* (*Pax3*) mutant; [141]), as might be expected if the two main cushions do not fuse and separate (Figure 6B). In contrast, three leaflets are more commonly seen in the setting of single trunk associated with pulmonary atresia, as has been suggested in the *Tbx1* mouse mutant [61], and are likely to result from loss of the SHF-derived tissue that would normally form the pulmonary trunk. However, there is considerable variation in the numbers of leaflets found in the setting of common arterial trunk, even those where the lineage origin of the defect is clear, showing that there is not a straightforward relationship between abnormalities of particular cell types and valve phenotype.

## 15. Bicuspid Valves and Congenital Heart Malformations

BAV is frequently found in combination with other left ventricular outflow tract malformations, including coarctation of the aorta (which is particularly common in Turner syndrome) ventricular septal defects and hypoplastic left heart syndrome (reviewed in [116]). It is reasonable to suggest that when malformations frequently occur together, they share a common aetiology. This could be due to the disruption of specific genes or signalling pathways, or to disruption of related developmental processes. For example, it has been speculated that defects in NCC could lead to a range of defects that include BAV, coarctation of the aorta, common arterial trunk and VSD [142,143,144,145]. As all the affected areas have major contributions from NCC, this seems a reasonable conclusion. 

Alternatively, some groupings of malformations could be sequences rather than syndromes, meaning that a primary abnormality leads to secondary consequences that are not directly linked to the primary insult. An example of this might be some sub-types of HLHS, where it is speculated that the primary defect specifically affects the mitral valve, with all of the other defects (ventricular hypoplasia, aortic stenosis/atresia and aortic hypoplasia) being secondary consequences of this that result from disrupted blood flow (the “no flow no grow” hypothesis (reviewed in [6]). It remains to be seen whether the association of left-sided ventricular outflow tract (LVOT) anomalies, that includes BAV, aortic stenosis, VSD and coarctation of the aorta and HLHS, is linked to a genetic defect that disrupts all of the affected areas of the heart, with variable expressivity. BAV has also been linked to atherosclerotic vascular disease as well as aortopathy and linked syndromes, e.g., Marfan, (reviewed in [116]). In all these cases it will be important to determine whether there is a common genetic pathway or progenitor cell linking the conditions or whether they are linked by physiological disturbance.

In contrast to BAV, isolated bicuspid pulmonary valve (BPV) is extremely rare, except in patients with tetralogy of Fallot, where it affects half to 2/3 of patients [146]. Surprisingly, the morphology of these valves has not been systematically described, probably because it has no bearing on the immediate surgical issues. However, as during surgical repair the transannular patch is placed on the accessible anterior aspect of the pulmonary trunk, it is possible that the anterior intercalated leaflet is missing in the majority of cases. Anecdotally, it has been suggested that raphes are not seen in these BPV. Clearly, an analysis of the morphology of BPV is needed. If the anterior intercalated leaflet is missing, this would support the notion that tetralogy of Fallot is due to an abnormality of the SHF. This is important since it was assumed for many years that the outflow defects seen in DiGeorge syndrome—most commonly tetralogy of Fallot or common arterial trunk—were due to a defect in NCC [147,148]. However, since the identification of *TBX1* as the main cardiovascular gene linked to DiGeorge syndrome, it has become clear that this is an indirect effect on NCC, as *TBX1* is not expressed in NCC, but it influences their migration (reviewed in [149,150,151]). Furthermore, three of the characteristic malformations found in tetralogy of Fallot (pulmonary atresia, deviation of the outlet septum and ventricular septal defect) can be linked to defects in the SHF [61,151,152], whilst the fourth, right ventricular hypertrophy does not manifest until after birth and may be secondary to the other defects.

## 16. The Genomics of BAV

Genomic analyses of congenital heart disease have been successful in discovering a small number of variants in the small percentage of patients with syndromic conditions [153,154,155,156]. However, the genetic basis of common conditions such as BAV, even when appearing to affect families, has remained elusive. A number of genes including *NOTCH1, GATA 4*, *5* and *6*, and have been previously implicated in CHD from mouse models [157,158,159,160,161,162,163,164,165,166]. Thus, there is obviously good reason to consider these genes as candidates for human BAV. However, knockout of most of these genes results in severe, complex cardiovascular defects, rather than simply BAV, and modelling of human variants of these genes is limited and often disappointing. This is particularly so with *NOTCH1*. Variants are readily reported in genomic studies, but mouse knock out studies do not recapitulate the BAV (see [127]). Some of this may relate to incomplete penetrance where the mice carry a variant but are not subject to malformation (as is commonly seen in BAV families) or variable expressivity where variations of phenotype are seen. If so, this questions the utility of functional assays in preclinical models, and also preventative treatment or preconceptual counselling for families. Alternatively, some variants may not be in themselves pathological in isolation but may interact with the individual’s developmental genetic landscape to make abnormalities more likely. If there is a specific modifier gene, we might call this oligogenic inheritance, but a range of modifiers may equally contribute. Again, in this scenario, it is very difficult to perform functional assays. There is also a problem regarding what functional assays to perform. The most popular functional assay for BAV variants is an EndMT assay. However, the failure of EndMT results in hypoplasia of the cushions (e.g., [167]) and this causes severe septal as well as valve defects (e.g., [168]). Hence, there is no experimental evidence to indicate a failure of EndMT could cause isolated BAV. Furthermore, EndMT is only one discrete process in valve development—much of which we still do not mechanistically understand. Another example of a suggested BAV-causing gene is *GATA4* (e.g., [163,169,170]). Patient variants in *GATA4* have been shown to disrupt EndMT in induced pluripotent stem cells [163] and *Gata4* has been shown to be required for EndMT to form the endocardial cushions in the mouse heart [26]; indeed, mice lacking functional *Gata4* have severe heart defects. Unfortunately, to our knowledge, BAV has not been reported in any mouse models of *Gata4* insufficiency (e.g., [171,172,173]). In contrast, *GATA6* seems more likely to be relevant to BAV. *GATA6* variants have been reported in patients with BAV [161,174,175] and these have been at least partially validated, in some cases by in vitro assays (showing that patient variants had reduced transcriptional activity; [174]) and importantly by knocking out *Gata6* in mice and showing that BAV results [161]. Of relevance to this review, it was also shown that knockout of *Gata6* solely in SHF progenitors was enough to recapitulate the BAV seen in total knockouts [161], highlighting the importance of this lineage in the development of the arterial valve leaflets. Studies using zebrafish have also been able to validate the importance of BAV variants for cardiovascular development (e.g., [100]), although these have the disadvantage of not being able to recapitulate the BAV phenotype itself, as zebrafish ordinarily have a bicuspid aortic valve. The best validated BAV gene currently is *ROBO4* [166]. A potential disease-causing variant in this gene was originally identified by whole exome sequencing in a family with BAV, although in the presence of thoracic aortic aneurysm. Further variants were then identified in another small family and in sporadic cases with a mixture of RL and RN fusions. The relevance of *ROBO4* for BAV was confirmed in a variety of ways. This included in vitro studies that showed that *ROBO4* variant alleles disrupt endothelial barrier function, a mouse knockout for *Robo4* that developed BAV and aortic aneurysm, and most impressively, a mouse knock-in of the specific variant that was identified in the original family, which also developed BAV and aortic aneurysm [166]. This comprehensively validates the *ROBO4* gene as a cause of BAV in the setting of aortopathy. Although this sets a “gold standard” for such studies it is again remarkable that a gene has been fully validated for a syndromic/familial condition rather than in sporadic cases. Finally, it may be time to re-evaluate the mechanisms by which sporadic developmental defects occur as arguably, the sum of experience to date suggests that the simple concept of the disease-causing gene variant is not valid. Other modes of disturbing genetic cascades such as alternative splicing or stochastic mechanisms may need to be explored.

## 17. Final Conclusions

In this review, we have indicated the major roles performed by NCC and the SHF in forming the arterial valves elucidated using animal models. In contrast the recent attempts to interpret the causes of human malformations through genomic approaches have been limited. It is clear that objective and detailed analysis of the arterial valve phenotypes in human aortic and pulmonary valve malformations has not been historically performed and this is especially relevant in bicuspid aortic and pulmonary valves. Similarly, in developmental studies there are areas of valve development, such as sculpting and sinus formation, that have received little attention so far, but are essential to understand if variants invoked in human disease conditions are to be correctly interpreted. Finally, the homeostatic biology of the valves is of great relevance for the aging human heart and in particular it will be essential to know whether developmental populations such as SHF and NCC, or indeed later additions from bone marrow-derived cells, have specific roles, or alternatively, the differentiated identity, regardless of origin, is what matters.
